# Can routine chest CT with artificial intelligence tool be used for opportunistically osteoporosis screening? A preliminary study

**DOI:** 10.3389/fmed.2026.1936400

**Published:** 2026-10-27

**Authors:** Runhai Zhang, Haohan Li, Ming Lu, Suping Chen, Caijun Huang

**Affiliations:** 1 Department of Radiology, Guiqian International Hospital, Guiyang, Guizhou, China; 2 CT Research Center, GE HealthCare, Changsha, China

**Keywords:** artificial intelligence, bone mineral density, chest CT, opportunistic screening, osteoporosis

## Abstract

**Introduction:**

Patients with pulmonary diseases face an elevated risk of osteoporosis. Conventional diagnosis relies on quantitative computed tomography (QCT), which delivers a high radiation dose and requires a dedicated scan. Whether conventional chest CT can be used for osteoporosis screening remains uncertain.

**Methods:**

A deep learning system was used to measure bone mineral density (BMD) in routine chest CT scans for osteoporosis screening, with QCT as the reference standard. A total of 98 patients who underwent chest and abdominal CT due to medical conditions between August 2024 and March 2025 were analyzed. The BMD of the thoracic vertebrae (T1-T12) was measured, three consecutive vertebral bodies were grouped into averages, and the results were compared with the BMD of the lumbar vertebrae (L1-L3) measured by standard QCT. The differences and agreement between the two BMD measurement methods were compared, and the diagnostic performance for osteoporosis and low bone mass was assessed by receiver operating characteristic curve analysis.

**Results:**

The cohort included 98 patients (48 female, 50 male; mean age 49.48 ± 14.64 years). AI-measured thoracic vertebrae BMD correlated highly with QCT BMD (r: 0.72-0.92), with the highest correlation observed for T10-T12 (r = 0.98, *P* < 0.001). All AI-BMD measurements demonstrated high diagnostic performance for osteoporosis and low bone mass. In the osteoporosis analysis,the T10-T12 BMD achieved the highest AUC of 0.998 (95% CI: 0.960-1.000), with an optimal diagnostic threshold of BMD ≤90.86 mg/cm³ (sensitivity 100.0%, specificity 97.6%). In the low-bone-mass analysis, the AUC was 0.990 (95% CI: 0.945-1.000). The volumetric CT dose index for QCT scans was significantly higher than that for chest CT scans (14.63 ± 2.30 vs. 4.13 ± 1.01 mGy, *P* < 0.001), and the post-processing time for QCT was significantly longer than that for the AI-based measurement (55.76 ± 9.05 vs. 7.50 ± 0.78 s, *P* < 0.001).

**Conclusion:**

AI-based BMD measurements from chest CT correlate highly with QCT measurements. AI-derived thoracic BMD from routine chest CT may serve as a potential opportunistic screening-support tool for osteoporosis and low bone mass, but further validation in larger, multicenter, prospective cohorts is required before clinical adoption.

## 1 Introduction

Various pulmonary diseases, such as chronic obstructive pulmonary disease and cystic fibrosis, along with glucocorticoid therapy for these conditions, elevate the risk of osteoporosis. Osteoporosis is a skeletal disorder characterized by diminished bone mass and a deterioration of bone microarchitecture (1, 2). Its most common and serious complication is fracture, which represents a major cause of disability and reduced mobility (3–5). Consequently, the simultaneous screening and evaluation for osteoporosis in patients with pulmonary diseases is a critical component of clinical prevention and intervention (6, 7).

Although dual-energy x-ray absorptiometry (DXA) has been widely adopted for bone mineral density (BMD) measurement, its application requires considerable time; furthermore, its diagnostic utility is limited to assessing two-dimensional planar bone mineral density, and its results can be adversely affected by degenerative changes, osteophytes, aortic calcification, and other factors. Quantitative Computed Tomography (QCT) is increasingly being adopted as a screening tool for osteoporosis, because it enables volumetric analysis, provides precise anatomical localization, and is relatively insensitive to confounding skeletal pathologies (8, 9). Current diagnostic criteria for individual osteoporosis, however, are largely based on the lumbar spine rather than the thoracic spine. Standard QCT protocols involve higher radiation exposure, and the analysis often depends on specialized CT equipment and complex post-processing. As a result, diagnosing osteoporosis in patients with thoracic conditions via QCT frequently requires an additional lumbar scan. Concerns regarding radiation dose and the frequently asymptomatic nature of osteoporosis contribute to the common oversight of BMD measurement in pulmonary disease patients.

In clinical practice, patients with pulmonary diseases routinely undergo chest CT scans for diagnostic purposes (10–12). The radiation dose for a chest CT, however, is typically lower than that for a lumbar QCT due to anatomical differences, which complicates opportunistic osteoporosis screening through QCT analysis. Advances in artificial intelligence have promoted research on using machine learning and deep convolutional neural networks to measure BMD and diagnose osteoporosis from low-dose chest CT images (13–16), offering a potentially simpler screening approach. However, most of these studies have focused on the lumbar spine or abdominal CT examinations, with relatively few studies exploring the value of thoracic vertebra BMD measured from chest CT for individual osteoporosis diagnosis (17, 18). This study aims to technically validate AI-based thoracic vertebral BMD measurement from routine chest CT as an opportunistic screening-support tool for osteoporosis and low bone mass.

## 2 Methods

### 2.1 Study population

Patient data were retrospectively derived from a prospective study of patients admitted for first-episode acute pancreatitis, registered with the Chinese Clinical Trial Registry (ChiCTR 2400082188), conducted in accordance with the Declaration of Helsinki (2013 revision), and approved by the Ethics Committee of Guiqian International Hospital (Approval Number: Guiqian 2024 Ethics Review No. 04). All methods were carried out in accordance with relevant guidelines and regulations.

From this retrospective cohort, 145 participants who had undergone both chest and abdominal CT examinations within a 7-day period between August 2024 and March 2025 were consecutively included. We excluded 47 participants with a history of malignant tumors or spinal surgery, images with significant artifacts, and other conditions precluding reliable BMD measurement by any method, such as compression fractures, severe scoliosis, or other vertebral abnormalities (Figure 1). A total of 98 participants were included in the final study cohort (48 female and 50 male; mean age 49.48 ± 14.64 years; range, 18–87 years).

**Figure 1:**
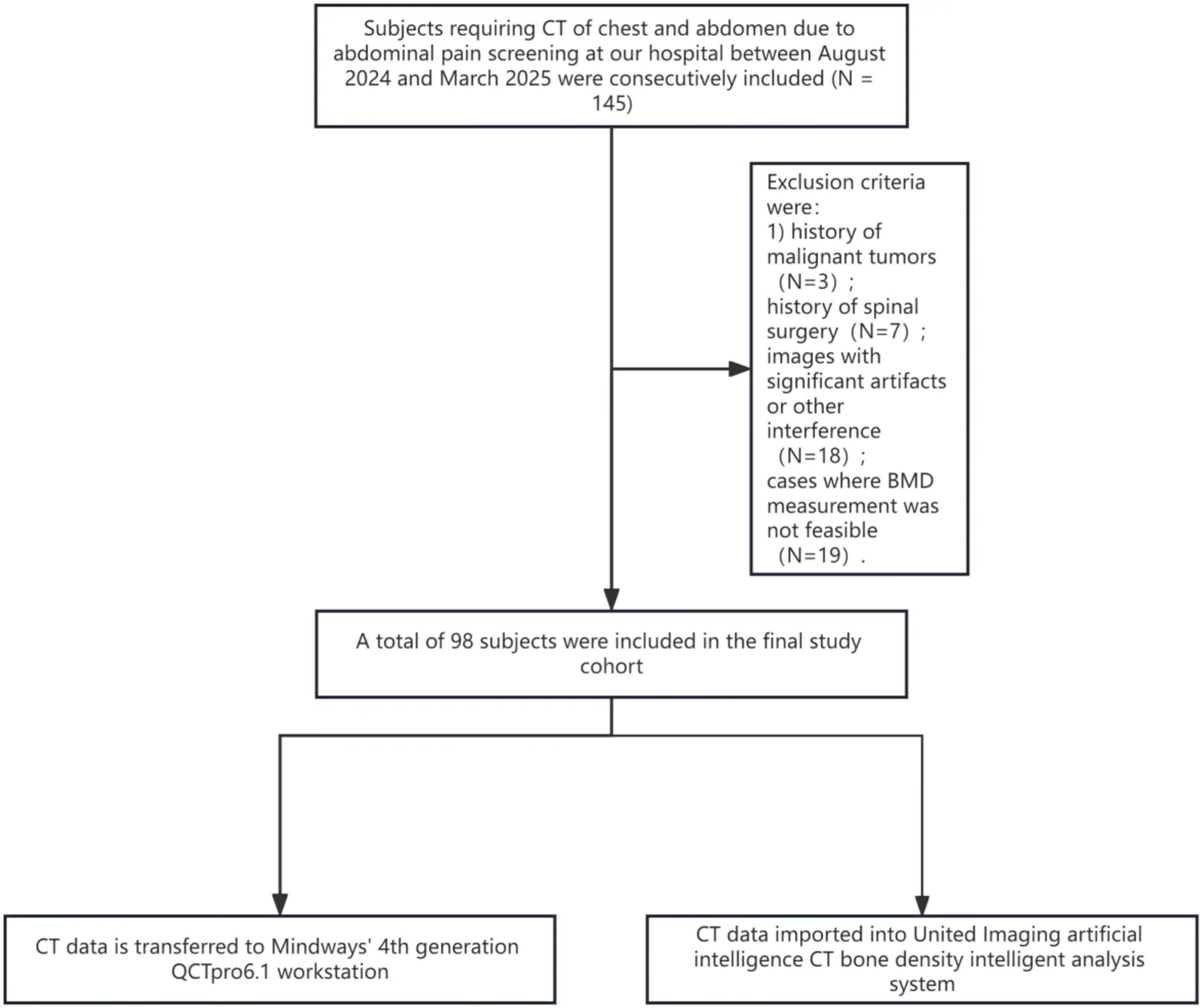
Flowchart of participant inclusion.

### 2.2 CT data acquisition

All patients presented with abdominal pain and underwent combined chest and abdominal CT scans within a seven-day period. All scans were acquired on a single 256-slice CT scanner (Revolution CT, GE HealthCare, USA) using 120 kVp tube voltage, Smart mA tube current, 5 mm slice thickness and 5 mm slice interval for the original acquisition, and a pitch of 0.992:1. Only non-contrast chest and abdominal CT images were included in this study. Images were reconstructed with 1.25 mm thickness, standard (Stnd) reconstruction kernel, helical plus mode, window width 400, window level 40, and 1.25 mm interval. AI-BMD analysis was performed using this standard reconstruction. The volumetric CT dose index (CTDIvol) of each scan was recorded. The chest scan extended from the lung apices to the 12th thoracic vertebra (T12), while the abdominal scan covered the entire pancreas.

### 2.3 QCT bone mineral density measurement

BMD measurements were performed using QCT (American Mindways Company, Austin), with daily phantom calibration performed prior to measurement and a standard reconstruction algorithm applied. CT data were analyzed on a Mindways 4th Generation QCT Pro workstation (version 6.1). BMD was measured within the cylindrical regions of interest (ROI), which were 9 mm high and placed in the center of L1 to L3 vertebral bodies avoiding cortical bone and basivertebral veins. The BMD values (mg/cm^3^) of L1 to L3 were recorded and analyzed, respectively, and the average was calculated. The total processing time was recorded. To ensure consistency, all BMD measurements were performed by one radiologist (R.Z.). A randomly selected subset of 30 samples was independently re-measured at a two-week interval by a second radiologist (H.L.) with more than 5 years of experience in musculoskeletal imaging, blinded to the initial results.

### 2.4 American college of radiology (ACR) QCT classification

Each participant bone status was categorized into normal (BMD >120 mg/cm^3^), osteopenia (80 mg/cm^3^ ≤ BMD ≤120 mg/cm^3^), and osteoporosis (BMD<80 mg/cm^3^) in accordance with the American College of Radiology QCT diagnostic criteria (19). Low BMD was defined as BMD ≤120 mg/cm^3^, including osteopenia and osteoporosis. Accordingly, patients were classified as normal (*n* = 61), osteopenia (*n* = 22), and osteoporosis (*n* = 15).

### 2.5 Artificial intelligence bone mineral density measurement

This study employed an artificial intelligence-based CT bone mineral density detection system utilizing deep learning and machine learning algorithms (Phantom-less Bone Mineral Density Calculation Software, uAI-Discover BMD, version R001, United Imaging Intelligence Co., Ltd., Shanghai, China). It corrects for scanner differences and factors affecting CT values (e.g., beam hardening) using stable internal references and other measurement parameters, thereby converting CT values into BMD values. After importing chest CT data, the software automatically segmented thoracic vertebrae (T1-T12) and measured BMD—mimicking the anatomical localization logic of lumbar QCT to ensure comparability between thoracic and lumbar BMD results. BMD was measured within the cylindrical ROI, which were 9 mm high and placed in the center of vertebral bodies avoiding cortical bone and basivertebral. The BMD values (mg/cm^3^) of T1 to L3 were recorded and analyzed, respectively, and the average was calculated. We recorded the time from software launch to the successful acquisition of thoracic BMD values. Subsequently, to reduce the variability in single vertebral measurements, and following the lumbar spine QCT measurement protocol, we exploratively calculated the mean values of the obtained bone mineral density data across every three consecutive vertebrae, thereby establishing four subgroups: T1–T3, T4–T6, T7–T9, and T10–T12. Notably, this software is a pre-trained, commercially available, regulatory-cleared system; no model training, fine-tuning, or optimization was performed on the present study data, and therefore there was no risk of data leakage into model development.

### 2.6 Statistical analysis

Statistical analyses were conducted with SPSS 27.0 and MedCalc® V22.001. Continuous variables were assessed for normality using the Shapiro–Wilk test. Normally distributed variables are presented as mean ± standard deviation (SD), and non-normally distributed variables are presented as median (interquartile range, IQR), while categorical variables are expressed as counts (percentages). Normally distributed paired data were compared using paired t-tests with Bonferroni correction for multiple comparisons. Agreement between AI-BMD and QCT-BMD was assessed via Bland-Altman analysis. Pearson correlation coefficients were used to assess the correlations between AI-BMD and both lumbar QCT-BMD and age. The diagnostic performance of AI-BMD for osteoporosis and low bone mass was evaluated using receiver operating characteristic (ROC) curve analysis. The Area Under the Curve (AUC) was calculated, and the cutoff threshold corresponding to the maximum Youden index was derived from the ROC curve to determine sensitivity and specificity. The 95% confidence intervals (CI) for the AUC were calculated using the non-parametric method of DeLong method. Internal validation was performed using bootstrap resampling (1000 iterations) with the rms R package validate() function (Harrell's method), and the optimism-corrected C-index was calculated, optimal thresholds, and Youden indices. Sensitivity and specificity at the optimal thresholds were reported with exact 95% CI, and positive predictive value (PPV), negative predictive value (NPV), accuracy, and F1-score were derived from the corresponding confusion matrices.

## 3 Results

### 3.1 Demographic information and BMD measurements of the vertebrae

Among the 98 patients, 61 had normal bone mass, 22 had osteopenia, and 15 had osteoporosis. In all patients, AI-BMD values showed a progressive decrease from T1 to L3, and thoracic BMD values were consistently higher than lumbar BMD values (all *P* < 0.001) (Table 1). Bone mineral density in both the thoracolumbar segments showed a significant negative correlation with age (*r* = −0.546 to −0.722, all *P* < 0.001). (Figure 2).

**Table 1:** Distribution and differences of demographics and BMD among the normal, osteopenia, and osteoporosis groups.

Variables	Total (*N* = 98)	Normal (*N* = 61)	Osteopenia (*N* = 22)	Osteoporosis (*N* = 15)	Statistic	*P*
Sex					6.15	0.046
Male	50 (51.0%)	31 (50.8%)	15 (68.2%)	4 (26.7%)		
Female	48 (49.0%)	30 (49.2%)	7 (31.8%)	11 (73.3%)		
Age (years)	49.48 ± 14.64	42.54 ± 11.39	56.41 ± 11.45	67.53 ± 9.92	35.390	<0.001
＜50	47 (48.0%)	42 (68.9%)	5 (22.7%)	0 (0.0%)	30.10	<0.001
≥50	51 (52.0%)	19 (31.1%)	17 (77.3%)	15 (100.0%)		
BMI (kg/m^2^)	23.76 ± 3.13	23.60 ± 3.28	23.88 ± 2.96	25.24 ± 1.31	0.52	0.599
T1	171.10 (140.68, 206.80)	198.20 (175.40,237.00)	145.10 (137.00,156.50)	110.60 (101.65,128.00)	67.58	<0.001
T2	165.10 (133.88,196.19)	184.80 (164.70,227.80)	137.80 (127.56,157.54)	105.10 (92.95,121.85)	55.79	<0.001
T3	162.22 (132.70,198.31)	180.90 (160.60,221.90)	140.05 (125.30,147.52)	104.50 (93.25,109.40)	54.35	<0.001
T4	157.90 (132.98,190.30)	179.90 (158.00,213.90)	136.35 (120.65,146.12)	97.70 (85.60,103.00)	55.53	<0.001
T5	154.35 (127.03,183.12)	176.40 (155.20,206.30)	127.47 (117.70,139.40)	91.30 (82.25,102.70)	63.81	<0.001
T6	152.30 (117.01,181.90)	168.90 (155.80,201.00)	119.03 (110.52,137.40)	84.90 (76.30,85.90)	66	<0.001
T7	146.50 (111.48,177.11)	166.80 (147.20,194.70)	114.30 (103.28,127.45)	82.10 (73.75,87.50)	65.32	<0.001
T8	141.20 (108.35,172.35)	168.85 (142.60,190.70)	114.75 (99.41,122.88)	76.50 (75.30,83.00)	66.7	<0.001
T9	145.75 (108.38,172.72)	164.00 (150.60,188.20)	112.05 (101.05,122.04)	82.10 (71.25,85.15)	68.25	<0.001
T10	142.15 (110.10,174.80)	169.70 (145.40,190.00)	111.18 (106.68,120.85)	80.90 (72.05,88.95)	67.65	<0.001
T11	141.75 (107.12,175.52)	169.80 (147.80,193.30)	107.60 (105.10,122.52)	79.00 (71.50,83.20)	69.06	<0.001
T12	137.55 (105.53,171.50)	168.10 (142.80,188.20)	106.70 (99.60,118.92)	74.60 (66.80,82.15)	68.89	<0.001
L1	133.10 (98.62,170.57)	163.30 (137.50,183.80)	101.62 (90.00,110.17)	70.30 (57.65,74.95)	70.36	<0.001
L2	129.75 (95.15,164.80)	156.30 (131.80,178.60)	98.85 (89.88,105.59)	66.50 (44.05,72.35)	70.4	<0.001
L3	122.50 (93.12,162.60)	151.60 (127.30,172.00)	95.80 (84.43,100.38)	58.10 (43.15,71.00)	69.16	<0.001
T1-T3	165.53 (138.12,199.77)	185.67 (168.30,229.90)	141.02 (129.13,156.41)	108.83 (97.40,121.42)	58.12	<0.001
T4-T6	156.35 (125.64,181.69)	174.15 (157.60,206.30)	126.77 (113.83,141.02)	91.53 (85.35,100.68)	63.59	<0.001
T7-T9	145.28 (108.81,173.33)	165.20 (149.80,190.95)	115.42 (101.81,122.49)	79.00 (75.20,87.60)	67.36	<0.001
T10-T12	141.67 (107.56,173.14)	171.03 (145.75,192.17)	107.87 (104.58,120.67)	78.70 (71.38,83.83)	69.85	<0.001
L1-L3	129.17 (97.48,164.50)	159.15 (131.37,176.47)	98.74 (89.25,103.14)	66.20 (49.58,72.30)	70.75	<0.001
QCT	130.30 (100.78,168.35)	162.80 (134.30,183.40)	102.45 (93.52,109.20)	66.60 (50.95,74.65)	72.17	<0.001

**Figure 2:**
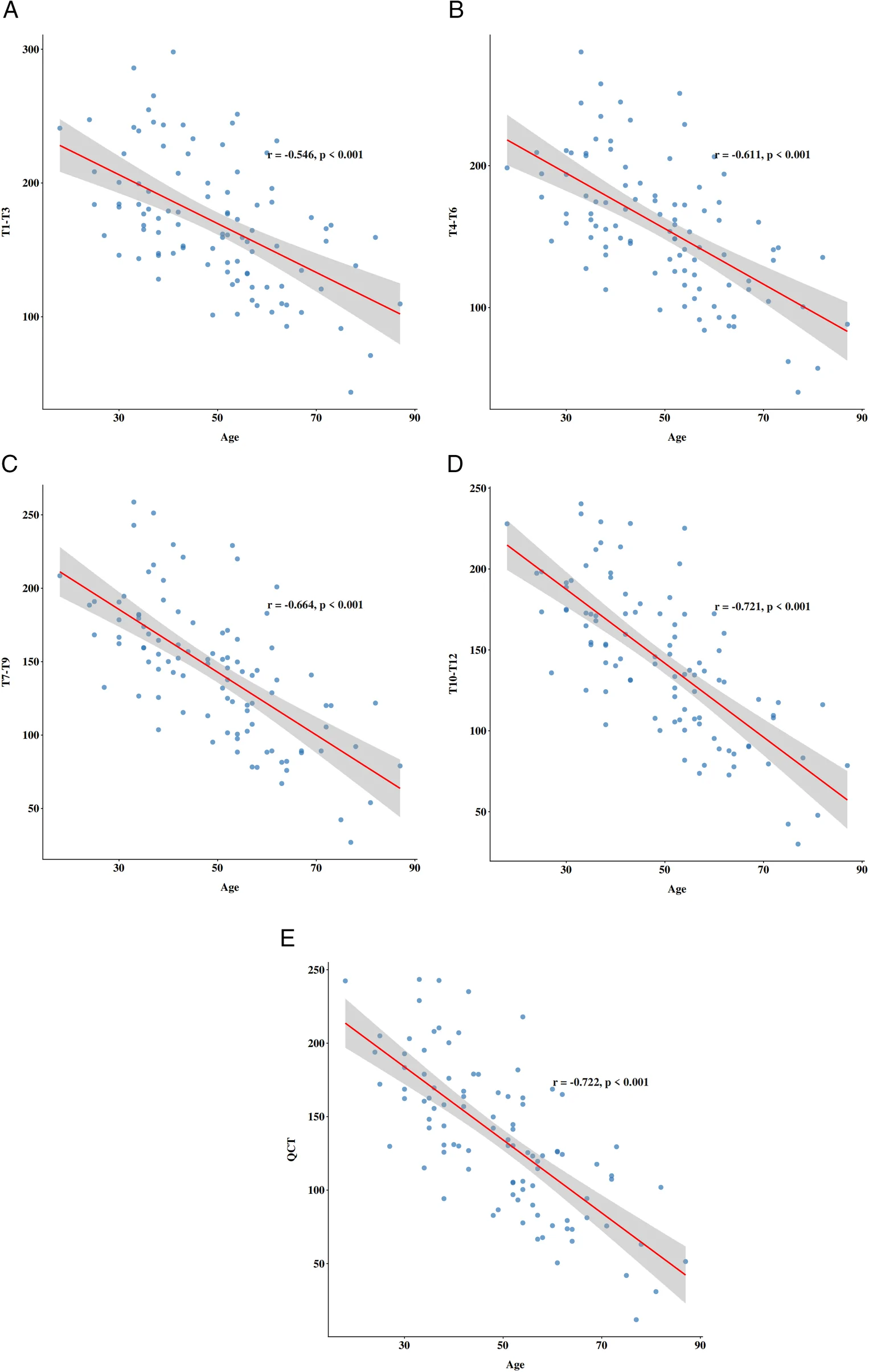
Correlation between age and BMD. **(A)** T1–T3; **(B)** T4–T6; **(C)** T7–T9; **(D)** T10–T12; **(E)** QCT.

Osteoporosis prevalence was higher in women than in men (11/48, 22.9% vs. 4/50, 8.0%; *P* = 0.046), and all patients with osteoporosis were aged ≥50 years (*P* < 0.001). BMI was significantly higher in men than in women (24.70 ± 2.97 vs. 22.73 ± 3.01 kg/m^2^, *P* = 0.007), but BMI did not differ significantly among the normal, osteopenia, and osteoporosis groups (23.60 ± 3.28 vs 23.88 ± 2.96 vs. 25.24 ± 1.31 kg/m^2^, *P* = 0.599), BMD values at all vertebral levels did not differ significantly by sex (all *P* > 0.05). Both AI-BMD and QCT-BMD values were significantly higher in the younger subgroup (*P* < 0.001) (Table 1). These subgroup results should be considered exploratory given the small sample size. Agreement was assessed using the intraclass correlation coefficient (ICC). The single-measure ICCs for L1, L2, and L3 were 0.998 (95% CI: 0.990–0.999), 0.994 (95% CI: 0.978–0.999), and 0.998 (95% CI: 0.995–1.000), respectively, and the average-measure ICC for L1–L3 was 0.998 (95% CI: 0.992–1.000), indicating excellent inter-observer agreement (Supplementary Table S3).

### 3.2 Differences and correlation between thoracic and lumbar BMD

The mean deviation of thoracic vertebral BMD (from T1 to T12) relative to lumbar vertebral BMD gradually decreased. Among the four thoracic vertebral groups, the T10-T12 group showed the best consistency with QCT-BMD(*r* = 0.98, *P* < 0.001); Bland-Altman analysis confirmed good agreement between T10-T12 BMD and QCT-BMD (mean deviation: 7.1 mg/cm^3^; 95% LOA: -13.9 to 28.1 mg/cm^3^), further supporting its potential utility as a screening tool for osteoporosis (Figure 3). All thoracic vertebral BMD values were strongly positively correlated with lumbar vertebral BMD (*r* = 0.88 to 0.98). The strongest correlations was T10–T12 group (*r* = 0.98). The full correlation matrix is presented as a heatmap (Figure 4).

**Figure 3:**
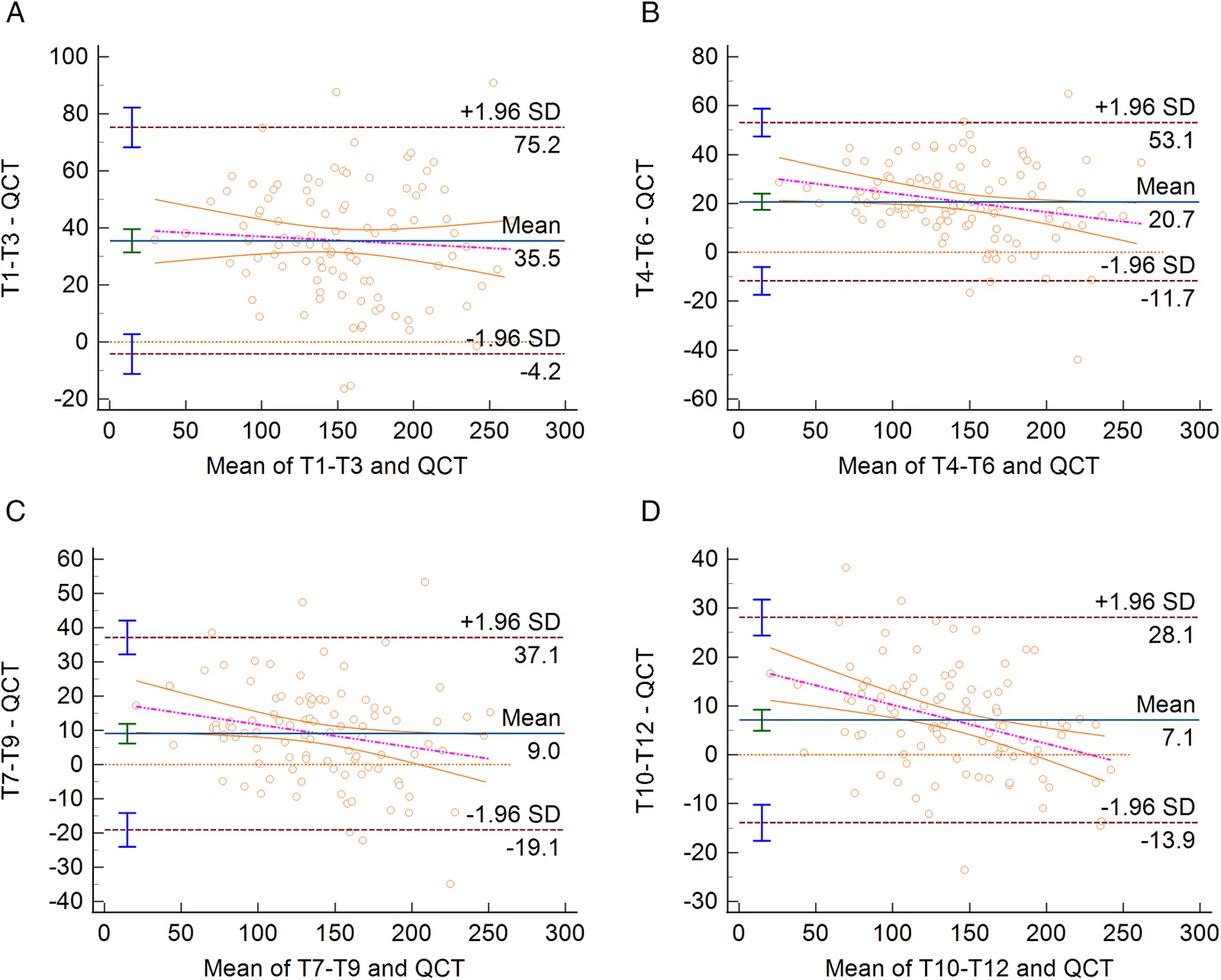
Bland-altman plots of AI-assessed thoracic BMD versus QCT-BMD. **(A)** T1–T3; **(B)** T4–T6; **(C)** T7–T9; **(D)** T10–T12.

**Figure 4:**
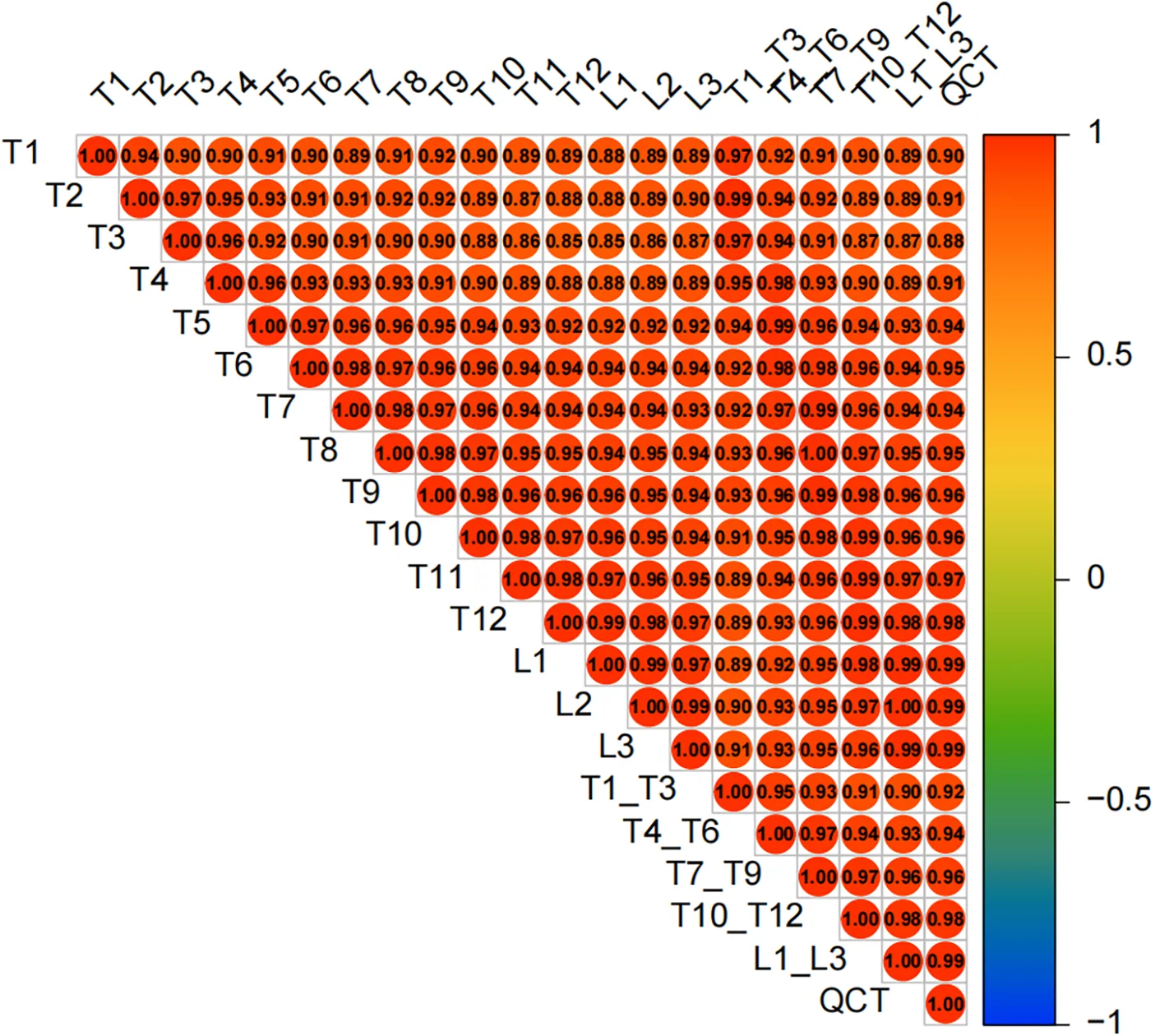
Correlation matrix heatmap of AI-measured BMD at individual vertebrae (T1–T12, L1–L3), three-vertebra averages (T1–T3, T4–T6, T7–T9, T10–T12, L1–L3), and QCT reference standard. Values represent Pearson correlation coefficients.

### 3.3 The diagnostic efficacy of AI-BMD for osteoporosis and low bone mass

Among the 98 included subjects, 15 were diagnosed with osteoporosis. ROC analysis demonstrated that the AUC values for the T10–T12 segments were superior to those of the other three segment groups(AUC = 0.998, 95% CI 0.960-1.000), with a sensitivity, speciﬁcity, NPV, PPV and accuracy of 100.0% (95% CI 78.2–100.0), 97.6% (95% CI 91.6–99.7), 100.00%, 88.24% and 98.00%, respectively (Figure 5). To further evaluate the predictive ability of T10-T12 without overﬁtting, the corrected AUC obtained by internal validation using the bootstrap method was 0.998 (95% CI 0.984-1.000), the calibration slope was 0.781; the calibration intercept was -0.455; and the Brier score was 0.016. In the low-bone-mass analysis, ROC analysis showed that T10–T12 also provided better AUC values than other three-vertebra groups (AUC = 0.990, 95% CI 0.945-1.000) with a sensitivity, speciﬁcity, NPV, PPV and accuracy of 97.30% (95% CI 85.8–99.9), 96.72% (95% CI 88.7–99.6), 98.33%, 94.74% and 96.94%, respectively. To further evaluate the predictive ability of T10-T12 without overﬁtting, the corrected AUC obtained by internal validation using the bootstrap method was 0.989(95% CI 0.961-0.998), the calibration slope was 0.870; the calibration intercept was -0.012; and the Brier score was 0.037 (Table 2). The more conservative AUC in the low-bone-mass analysis likely reflects the true discriminative ability more accurately, given the larger number of positive cases. Borderline cases (BMD within ±5 mg/cm^3^ of the threshold) accounted for both false positives in the osteoporosis analysis, while all true osteoporosis cases had BMD values clearly below the threshold. These findings indicate that misclassification occurs mainly near the threshold and that the method has a high NPV. Vertebral-level-specific analyses for both osteoporosis and low bone mass are provided in Supplementary Tables S1, S2. Diagnostic performance increased caudally, with T12 showing the closest correlation with lumbar QCT (*r* = 0.98) and excellent diagnostic accuracy (osteoporosis: AUC 0.998; low bone mass: AUC 0.987). For osteoporosis, no pairwise differences reached statistical significance (all *P* > 0.05), although T7–T9 (*P* = 0.057) and T10–T12 (*P* = 0.063) showed a trend toward higher AUC than T1–T3. For low bone mass, T7–T9 and T10–T12 were both significantly superior to T1–T3 (*P* = 0.030 and *P* = 0.036, respectively), whereas no significant differences were observed among T4–T6, T7–T9, and T10–T12. These findings indicate that diagnostic performance improved caudally and plateaued at T7–T12.

**Table 2:** Receiver operating characteristic curves for distinguishing osteoporosis in each group.

Group	AUC (95%CI）	Bootstrap AUC (95%CI）	Cut-off (mg/cm^3^)	Sensitivity% (95%CI)	Specificity% (95%CI)	PPV (%)	NPV (%)	Accuracy (%)	F1	Youden index
Low bone mass (LBM)										
T1-T3	0.944 (0.877–0.980)	0.943（0.875–0.975）	143.5	75.68 (58.8–88.2)	98.36 (91.2–100.0)	96.55	86.96	89.80	0.849	0.741
T4-T6	0.963 (0.904–0.991)	0.963 （0.903–0.987）	136.9	83.78 (68.0–93.8)	96.72 (88.7–99.6)	93.94	90.77	91.84	0.857	0.886
T7-T9	0.980 (0.928–0.998)	0.977 (0.935–0.994)	126.6	91.89 (78.1–98.3)	95.08 (86.3–99.0)	91.89	95.08	93.88	0.919	0.870
T10-T12	0.990 (0.945–1.000)	0.989 （0.961–0.998）	126.0	97.30 (85.8–99.9)	96.72 (88.7–99.6)	94.74	98.33	96.94	0.909	0.960
Osteoporosis										
T1-T3	0.970 (0.914–0.994)	0.953 (0.927–0.994)	138.2	100.00 (78.2–100.0)	86.75 (77.5–93.2)	57.69	100.00	88.78	0.732	0.897
T4-T6	0.991 (0.947–1.000)	0.991 (0.956–1.000)	104.4	93.33 (68.1–99.8)	98.80 (93.5–100.0)	93.33	98.80	97.96	0.933	0.921
T7-T9	0.996 (0.955–1.000)	0.995 (0.973–1.000)	92.17	100.00 (78.2–100.0)	97.59 (91.6–99.7)	88.24	100.00	97.96	0.938	0.976
T10-T12	0.998 (0.960–1.000)	0.998 (0.984–1.000)	90.86	100.00 (78.2–100.0)	97.60 (91.6–99.7)	88.24	100.00	98.00	0.938	0.976

BMD, bone mineral density; AUC, area under the curve; CI, confidence interval; PPV, positive predictive value; NPV: negative predictive value;low bone mass = osteopenia + osteoporosis.

**Figure 5:**
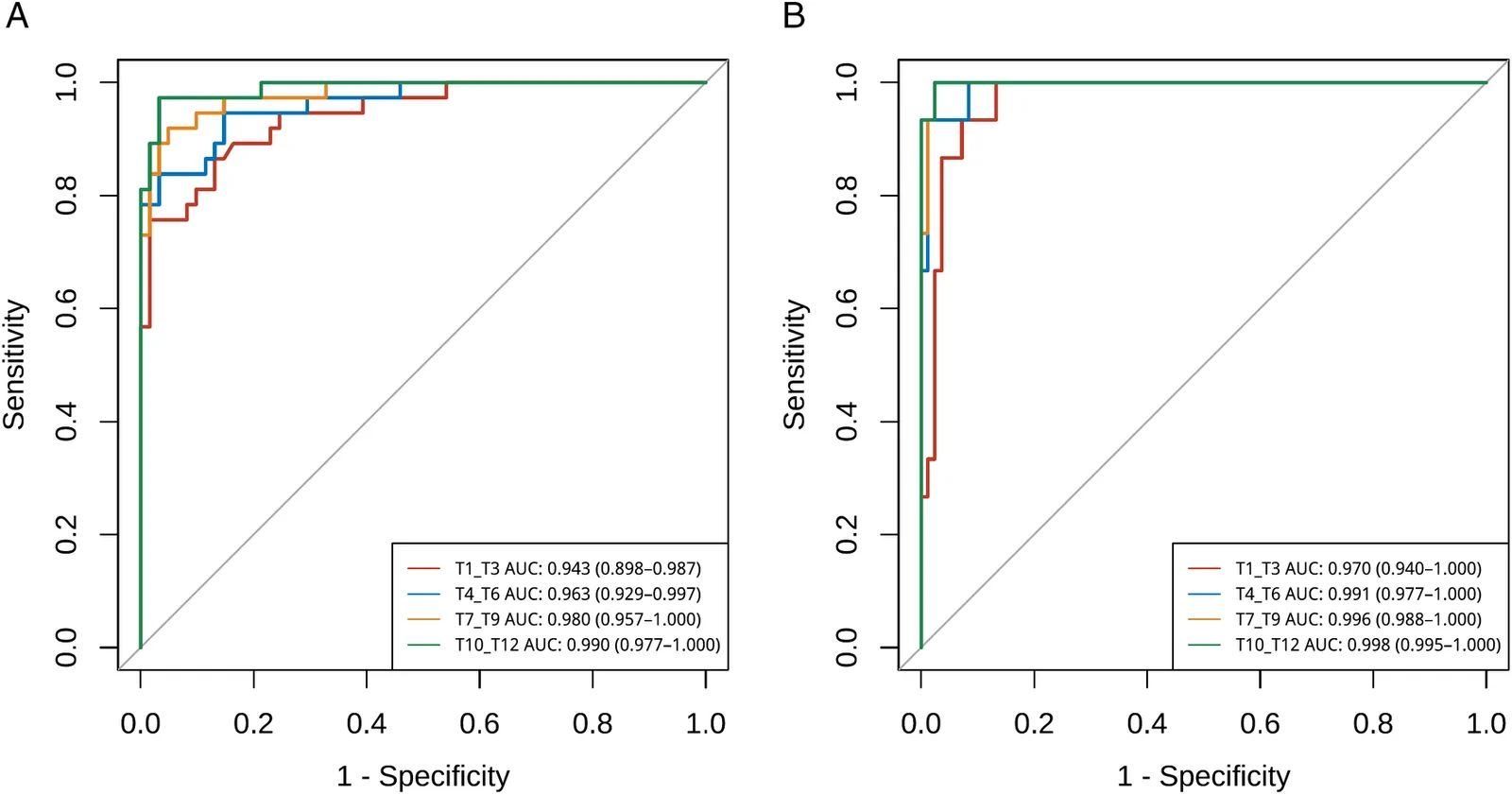
Receiver operating characteristic (ROC) curves for distinguishing low bone mass **(A)** and osteoporosis **(B)** in each three-vertebra group (T1–T3, T4–T6, T7–T9, T10–T12).

In addition, the CT dose index (CTDIvol) of QCT scans was significantly higher than that of chest CT scans (14.63 ± 2.30 vs. 4.13 ± 1.01 mGy, *P* < 0.001); the post-processing time of QCT was significantly longer than the AI-based BMD measurement time of chest CT (55.76 ± 9.05 vs. 7.50 ± 0.78 s, *P* < 0.001).

## 4 Discussion

This study is a technical validation of an AI-based phantom-less BMD measurement tool in a cohort of patients with acute pancreatitis who underwent both chest and abdominal CT. This cohort was selected because patients with pulmonary diseases typically undergo chest CT only and rarely have paired abdominal CT for QCT reference. Our results suggest that AI-based thoracic vertebral BMD measurement on routine chest CT may serve as a promising tool for opportunistic osteoporosis screening, addressing limitations of traditional methods such as radiation exposure and low efficiency. However, these findings are preliminary and require validation in pulmonary-disease and general screening populations before broader clinical application. In our cohort, thoracic vertebral BMD was consistently higher than lumbar BMD measured. A gradual decrease in BMD was observed from the T1 to T12 vertebral bodies, aligning with findings reported by George Simion et al. (9, 20). This trend likely relates to differences in vertebral stress. The sagittal curvature of the spine is known to directly influence load distribution (21), and variations in the load-strength ratio (LSR) can further alter stress distribution (22), ultimately leading to regional differences in BMD. From the cervical to the lower lumbar segments, increasing mechanical stress correlates with decreasing BMD, which helps explain the higher fracture incidence observed in the lower thoracic and lumbar regions (22). Given the more comparable mechanical loading conditions between the lower thoracic and lumbar spine, the AI-BMD values for the T10-T12 group are numerically closer to and show a stronger correlation with those of the L1-L3 group. The AI-BMD derived from T10-T12 demonstrates excellent diagnostic efficacy for lumbar osteoporosis (AUC = 0.998, *P* < 0.001). This performance is superior to that of other thoracic segment groups, particularly compared to the T1-T3 and T4-T6 group. Three consecutive vertebrae were averaged for two reasons: (1) to reduce the impact of individual vertebral abnormalities (e.g., compression fractures, implants, or degenerative changes) that may preclude reliable BMD measurement at a single level, consistent with standard QCT practice; and (2) Three-vertebra averaging improved correlation with QCT compared with individual vertebrae (T10–T12: *r* = 0.98 vs. T10: *r* = 0.96, T11: *r* = 0.97, T12: *r* = 0.98). These findings are consistent with previously reported by Ya Ling Pan et al. (23). Moreover, the vertebral-level-specific analysis demonstrated a caudal gradient of diagnostic performance for osteoporosis(AUC = 0.954-0.998), with the individual T12 vertebra showing the closest correlation with lumbar QCT (*r* = 0.98), further supporting the biomechanical similarity between the lower thoracic spine and the lumbar region. The 100% NPV of the T10-T12 measurement is a particularly favorable property for an opportunistic case-finding tool, for which reliably excluding osteoporosis is the key requirement; the few false positives would be clarified by confirmatory QCT in the proposed clinical workflow.

Although the AI-BMD of the T10-T12 group demonstrated high diagnostic efficacy for osteoporosis, its diagnostic threshold requires adjustment. In this study, a BMD threshold of ≤ 90.86 mg/cm^3^ yielded a sensitivity of 100% (95% CI 78.2–100.0) and a specificity of 97.6% (95% CI:91.6–99.7) for diagnosing osteoporosis in the T10-T12 group (Table 2); the corresponding confusion matrix yielded a PPV of 88.24%, an NPV of 100.00%, an accuracy of 98.00%, and an F1-score of 0.938. In 2021, Maximilian T Löffler et al. (24) proposed an artificial intelligence-based method, which achieved a slightly lower diagnostic efficacy for osteoporosis (AUC=0.86) than the present study. Furthermore, Sebastian Rühling et al. (25) established thoracic vertebral BMD thresholds for osteoporosis diagnosis from the cervical to lower thoracic vertebral bodies, proposing a general thoracic threshold of 100 mg/cm^3^ and a specific threshold of 83.8 mg/cm^3^ for the T12 vertebral body. These published values are broadly consistent with our findings, though slightly lower than the threshold identified here. The discrepancy likely arises from two methodological differences. First, our analysis employed the average bone mineral density (BMD) across three vertebrae (T10-T12), which is inherently higher than the BMD of a single T12 vertebra. Second, the threshold established here was validated against the QCT gold standard, whereas the prior threshold was derived solely from correlation analysis without such validation.

Both AI-BMD and QCT-BMD exhibited a strong negative correlation with age, a finding consistent with prior reports (25–27). Age-related declines in overall bone mineral density are well-documented, with osteoporosis being more prevalent in women. This increased prevalence is linked to factors including mineral loss and hormonal changes. The patient cohort in this study reflected this trend, with a higher proportion of female osteoporosis cases (11/15, 73.3%), but all osteoporosis cases were diagnosed in individuals over 50 years old, the diagnostic performance of the AI bone mineral density threshold in younger populations should be interpreted with caution.

In this study, the radiation dose for chest CT scanning was significantly lower, measuring only 28% of that for abdominal CT. This difference enables patients with pulmonary diseases to undergo both pulmonary diagnosis and osteoporosis assessment in a single CT examination, thereby avoiding the additional radiation exposure of a dedicated lumbar QCT scan. Furthermore, the AI-assisted diagnostic system automatically segments vertebral bodies and rapidly calculates BMD. Relative to manual QCT post-processing, this system reduces processing time by 87%, and its automated segmentation significantly improves inter-observer consistency by eliminating examiner subjectivity. Consequently, deep learning-based BMD measurement from chest CT scans may optimize osteoporosis prevention and management in this patient population. In addition, 19 patients were excluded because reliable BMD measurement was not feasible by any method, including QCT and AI, due to multiple compression fractures, severe scoliosis, or other vertebral abnormalities. In clinical practice, it is recommended to use the average value of adjacent vertebrae as a surrogate indicator, a approach also consistently adopted in this study. From a practical perspective, AI-BMD can be derived from already-acquired chest CT without additional scanning, radiation, or acquisition time, with an automated post-processing time of only 7.50 ± 0.78 s per case, making this approach feasible for large-scale opportunistic case-finding. The vertebral-level results further suggest that single-vertebra measurement at T12 may suffice, simplifying clinical implementation. Positive AI-BMD findings should nevertheless trigger confirmatory QCT or DXA before any treatment decision. DXA remains the clinical standard for osteoporosis diagnosis; however, DXA (areal BMD) and QCT (volumetric BMD) are not interchangeable, and DXA was not systematically available in our cohort. Validation of AI-BMD against DXA-defined outcomes, including fracture prediction, represents an important direction for future research.

This study has several limitations. First, The study population consisted of patients with first-episode acute pancreatitis rather than a population-based screening cohort, which may introduce selection and spectrum bias and limit the generalizability of our findings to routine opportunistic osteoporosis screening, therefore, the generalizability of our findings to these populations requires further validation. Second, This study used a single scanner model, a fixed tube voltage (120 kVp), and a single reconstruction protocol; therefore, the robustness of the AI system across different scanners, tube voltages, slice thicknesses, and reconstruction algorithms remains to be determined, and further multicenter validation is warranted. Third, Only non-contrast chest and abdominal CT images were included; therefore, the potential effect of intravenous contrast on AI-derived BMD estimation and osteoporosis classification could not be evaluated, and further studies are needed to determine whether contrast-enhanced CT affects measurement accuracy. Fourth, Other potential confounding factors, including menopausal status, corticosteroid use, anti-osteoporotic treatment, diabetes, renal disease, and vertebral fractures, were not consistently available and could not be incorporated into subgroup or multivariable analyses. In addition, because all osteoporosis cases were aged ≥50 years, the performance of AI-BMD in younger populations could not be assessed. Fifth, A formal robustness or failure-analysis study of the AI system in challenging cases (e.g., motion or metal artifacts, vertebral compression fractures, degenerative changes, osteophytes, vascular calcification, or incomplete vertebral visualization) was not performed, and future work should evaluate the AI system in these cases.

## 5 Conclusions

The measurement of thoracic vertebral BMD using chest CT combined with deep learning algorithms may serve as a potential opportunistic case-finding or screening-support tool for osteoporosis and low bone mass. T10–T12 BMD measurements are comparable to lumbar BMD. However, given the small sample size, medically selected population, lack of external validation, and limited assessment of scanner/protocol variability, this approach should not yet be considered a replacement for conventional osteoporosis assessment. Larger multicenter, prospective, and externally validated studies are warranted before clinical adoption.

## Data Availability

The raw data supporting the conclusions of this article will be made available by the authors, without undue reservation.
