# Supplementary material for: Can routine chest CT with artificial intelligence tool be used for opportunistically osteoporosis screening? A preliminary study
Source: Front Med (Lausanne). 2026 Oct 27;13:1936400. doi: 10.3389/fmed.2026.1936400 (PMC13750144; doi:10.3389/fmed.2026.1936400)
Supplement: Supplementary file 1 [file Table1.docx]

TableS1. Performance characteristics of level vertebral assessment for distinguishing low bone mass (osteopenia + osteoporosis)

| **Vertebrae** | **AUC(95%CI)** | **Cut-off**  **(mg/cm³)** | **Sensitivity**  **(95% CI)** | **Specificity (95% CI)** | **Youden index** | ***P*** |
| --- | --- | --- | --- | --- | --- | --- |
| T1 | 0.942 (0.899–0.985) | 161.85 | 0.919  (0.781,0.983) | 0.869  (0.758,0.942) | 0.788 | <0.001 |
| T2 | 0.933 (0.884–0.982) | 142.85 | 0.784  (0.618,0.902) | 0.967  (0.887,0.996) | 0.751 | <0.001 |
| T3 | 0.922 (0.861–0.984) | 149.35 | 0.865  (0.712,0.955) | 0.934  (0.841,0.982) | 0.799 | <0.001 |
| T4 | 0.929 (0.876–0.983) | 154.7 | 0.919  (0.781,0.983) | 0.820  (0.700,0.906) | 0.739 | <0.001 |
| T5 | 0.965 (0.932–0.997) | 132.15 | 0.811  (0.648,0.92) | 0.984  (0.912,1.000) | 0.795 | <0.001 |
| T6 | 0.973 (0.948–0.997) | 140.6 | 0.946  (0.818,0.993) | 0.885  (0.778,0.953) | 0.831 | <0.001 |
| T7 | 0.971 (0.943–0.998) | 142.15 | 0.973  (0.858,0.999) | 0.869  (0.758,0.942) | 0.842 | <0.001 |
| T8 | 0.976 (0.951–1.000) | 128.85 | 0.919  (0.781,0.983) | 0.951  (0.863,0.99) | 0.870 | <0.001 |
| T9 | 0.986 (0.968–1.000) | 124.625 | 0.892  (0.746,0.970) | 1.000  (0.941,1.000) | 0.892 | <0.001 |
| T10 | 0.982 (0.961–1.000) | 132.2 | 0.946  (0.818,0.993) | 0.93  (0.841,0.982) | 0.880 | <0.001 |
| T11 | 0.986 (0.972–1.000) | 130.2 | 0.973  (0.858,0.999) | 0.934  (0.841,0.982) | 0.907 | <0.001 |
| T12 | 0.987  (0.97–1.000) | 126 | 0.946  (0.818,0.993) | 0.951  (0.863,0.99) | 0.897 | <0.001 |
| L1 | 0.993 (0.982–1.000) | 118.65 | 0.973  (0.858,0.999) | 0.967  (0.887,0.996) | 0.940 | <0.001 |
| L2 | 0.992 (0.983–1.000) | 117.6 | 0.973  (0.858,0.999) | 0.934  (0.841,0.982) | 0.907 | <0.001 |
| L3 | 0.989 (0.975–1.000) | 115.95 | 1.000  (0.905,1.000) | 0.918  (0.819,0.973) | 0.918 | <0.001 |
