# Supplementary material for: Can routine chest CT with artificial intelligence tool be used for opportunistically osteoporosis screening? A preliminary study
Source: Front Med (Lausanne). 2026 Oct 27;13:1936400. doi: 10.3389/fmed.2026.1936400 (PMC13750144; doi:10.3389/fmed.2026.1936400)
Supplement: Supplementary file 2 [file Table2.docx]

TableS2. Performance characteristics of level vertebral assessment for distinguishing osteoporosis

| **Vertebrae** | **AUC(95%CI)** | **Cut-off**  **(mg/cm³)** | **Sensitivit%**  **(95% CI)** | **Specificit%**  **(95% CI)** | **Youden index** | ***P*** |
| --- | --- | --- | --- | --- | --- | --- |
| T1 | 0.954 (0.913–0.996) | 136.0 | 0.933  (0.681,0.998) | 0.928  (0.849,0.973) | 0.861 | <0.001 |
| T2 | 0.964 (0.93–0.998) | 140.1 | 1.000  (0.782,1.000) | 0.831  (0.733,0.905) | 0.831 | <0.001 |
| T3 | 0.972 (0.939–1.000) | 126.9 | 0.933  (0.681,0.998) | 0.928  (0.849,0.973) | 0.861 | <0.001 |
| T4 | 0.97 (0.923–1.000) | 125.1 | 0.933  (0.681,0.998) | 0.928  (0.849,0.973) | 0.861 | <0.001 |
| T5 | 0.99 (0.977–1.000) | 111.5 | 1.000  (0.782,1.000) | 0.964  (0.898,0.992) | 0.964 | <0.001 |
| T6 | 0.998 (0.995–1.000) | 100.8 | 1.000  (0.782,1.000) | 0.988  (0.935,1.000) | 0.988 | <0.001 |
| T7 | 0.994 (0.983–1.000) | 91.4 | 0.933  (0.681,0.998) | 1.000  (0.957,1.000) | 0.933 | <0.001 |
| T8 | 0.998 (0.993–1.000) | 92.1 | 1.000  (0.782,1.000) | 0.976  (0.916,0.997) | 0.976 | <0.001 |
| T9 | 0.989 (0.973–1.000) | 96.9 | 1.000  (0.782,1.000) | 0.964  (0.898,0.992) | 0.964 | <0.001 |
| T10 | 0.993 (0.982–1.000) | 101.7 | 1.000  (0.782,1.000) | 0.964  (0.898,0.992) | 0.964 | <0.001 |
| T11 | 0.998 (0.995–1.000) | 95.8 | 1.000  (0.782,1.000) | 0.976  (0.916,0.997) | 0.976 | <0.001 |
| T12 | 0.998 (0.994–1.000) | 91.6 | 1.000  (0.782,1.000) | 0.976  (0.916,0.997) | 0.976 | <0.001 |
| L1 | 0.997 (0.99–1.000) | 83.8 | 1.000  (0.782,1.000) | 0.988  (0.935,1.000) | 0.988 | <0.001 |
| L2 | 0.999 (0.997–1.000) | 83.6 | 1.000  (0.782,1.000) | 0.988  (0.935,1.000) | 0.988 | <0.001 |
| L3 | 0.991 (0.977–1.000) | 86.4 | 1.000  (0.782,1.000) | 0.928  (0.849,0.973) | 0.928 | <0.001 |
