# Supplementary material for: Can routine chest CT with artificial intelligence tool be used for opportunistically osteoporosis screening? A preliminary study
Source: Front Med (Lausanne). 2026 Oct 27;13:1936400. doi: 10.3389/fmed.2026.1936400 (PMC13750144; doi:10.3389/fmed.2026.1936400)
Supplement: Supplementary file 3 [file Table3.docx]

## Table S3.Interobserver reproducibility of QCT measurements

| Parameter | Observer 1 (mean±SD) | Observer 2 (mean±SD) | ICC(95% CI) |
| --- | --- | --- | --- |
| L1(mg/cm³) | 112.81±40.75 | 116.76±39.93 | 0.998(0.990-0.999) |
| L2(mg/cm³) | 109±42.85 | 112.13±41.67 | 0.994(0.978-0.999) |
| L3(mg/cm³) | 101.47±44.34 | 102.41±44.02 | 0.998(0.995-1.000) |
| L1-3(mg/cm³) | 107.76±42.44 | 110.43±41.61 | 0.998(0.992-1.000) |
| QCT measurement time (S) | 58.01±12.64 | 55.755±9.05 | 0.785(0.347-0.942) |
