# Supplementary material for: Can routine chest CT with artificial intelligence tool be used for opportunistically osteoporosis screening? A preliminary study
Source: Front Med (Lausanne). 2026 Oct 27;13:1936400. doi: 10.3389/fmed.2026.1936400 (PMC13750144; doi:10.3389/fmed.2026.1936400)
Supplement: Supplementary file 4 [file Table4.docx]

**Diagnosis of low bone mass using Delong test**

| Variable | AUC difference(95% CI) | Z | *P* |
| --- | --- | --- | --- |
| T1-T3 vs T4-T6 | -0.021 (-0.055,0.014) | -1.166 | 0.243 |
| T1-T3 vs T7-T9 | -0.037 (-0.070,-0.004) | -2.170 | **0.030** |
| T1-T3 vs T10-T12 | -0.048 (-0.092,-0.003) | -2.094 | **0.036** |
| T4-T6 vs T7-T9 | -0.016 (-0.047,0.014) | -1.050 | 0.294 |
| T4-T6 vs T10-T12 | -0.027 (-0.060,0.006) | -1.623 | 0.105 |
| T7-T9 vs T10-T12 | -0.011 (-0.029,0.008) | -1.120 | 0.263 |

**Delong test for osteoporosis diagnosis**

| Variable | AUC difference(95% CI) | Z | *P* |
| --- | --- | --- | --- |
| T1_T3 vs T4_T6 | -0.021 (-0.045,0.003) | -1.696 | 0.090 |
| T1_T3 vs T7_T9 | -0.026 (-0.052,0.001) | -1.906 | **0.057** |
| T1_T3 vs T10_T12 | -0.028 (-0.058,0.002) | -1.859 | **0.063** |
| T4_T6 vs T7_T9 | -0.005 (-0.020,0.010) | -0.628 | 0.530 |
| T4_T6 vs T10_T12 | -0.007 (-0.021,0.007) | -1.010 | 0.312 |
| T7_T9 vs T10_T12 | -0.002 (-0.009,0.004) | -0.753 | 0.451 |
